# Binding and structural analyses of potent inhibitors of the human Ca^2+^/calmodulin dependent protein kinase kinase 2 (CAMKK2) identified from a collection of commercially-available kinase inhibitors

**DOI:** 10.1038/s41598-019-52795-1

**Published:** 2019-11-11

**Authors:** Gerson S. Profeta, Caio V. dos Reis, André da S. Santiago, Paulo H. C. Godoi, Angela M. Fala, Carrow I. Wells, Roger Sartori, Anita P. T. Salmazo, Priscila Z. Ramos, Katlin B. Massirer, Jonathan M. Elkins, David H. Drewry, Opher Gileadi, Rafael M. Couñago

**Affiliations:** 1 Centro de Química Medicinal (CQMED), Centro de Biologia Molecular e Engenharia Genética (CBMEG), Universidade Estadual de Campinas (UNICAMP), Campinas, SP 13083-875 Brazil; 2 Structural Genomics Consortium, Departamento de Genética e Evolução, Instituto de Biologia, UNICAMP, Campinas, SP 13083-886 Brazil; 3 Structural Genomics Consortium, University of Oxford, Old Road Campus Research Building, Roosevelt Drive, Oxford, OX3 7DQ UK; 4 Structural Genomics Consortium, UNC Eshelman School of Pharmacy, University of North Carolina at Chapel Hill, Chapel Hill, NC 27599 USA

**Keywords:** Biochemistry, Structural biology

## Abstract

Calcium/Calmodulin-dependent Protein Kinase Kinase 2 (CAMKK2) acts as a signaling hub, receiving signals from various regulatory pathways and decoding them via phosphorylation of downstream protein kinases - such as AMPK (AMP-activated protein kinase) and CAMK types I and IV. CAMKK2 relevance is highlighted by its constitutive activity being implicated in several human pathologies. However, at present, there are no selective small-molecule inhibitors available for this protein kinase. Moreover, CAMKK2 and its closest human homolog, CAMKK1, are thought to have overlapping biological roles. Here we present six new co-structures of potent ligands bound to CAMKK2 identified from a library of commercially-available kinase inhibitors. Enzyme assays confirmed that most of these compounds are equipotent inhibitors of both human CAMKKs and isothermal titration calorimetry (ITC) revealed that binding to some of these molecules to CAMKK2 is enthalpy driven. We expect our results to advance current efforts to discover small molecule kinase inhibitors selective to each human CAMKK.

## Introduction

The Calcium/calmodulin-dependent kinases (CAMKs) respond to increases in the intracellular concentration of Ca^2+^ and play essential roles in several cellular processes, mainly via the activation of transcription factors. These enzymes share a modular architecture composed of a kinase domain (KD) followed by an auto-inhibitory sequence and a partially overlapping calmodulin-binding domain (CBD). CAMKs are usually kept in an inactive state through auto-inhibition, which can be relieved, upon an increase in Ca^2+^ levels, through the interaction of the protein CBD with Ca^2+^/calmodulin^1^. CAMK family members include CAMK1-4 and two CAMK kinases (CAMKK1-2). As mediators of the second messenger effects of Ca^2+^, CAMK family members play prominent roles in cell division (CAMK3 aka eEF-2K - eukaryotic elongation factor 2 kinase), neuronal development (CAMK1, 2 and 4) and immune response (CAMK4)^2,3^.

CAMK kinases (CAMKK) 1 and 2 are Ser/Thr kinases and upstream regulators of CAMKs^4–6^. For example, CAMKKs phosphorylate and activate CAMK1 and CAMK4, resulting in the activation of cyclic AMP-responsive element-binding protein (CREB). Consequently, both CAMKKs participate in several processes within the nervous system, such as neurite elongation and branching, long-term potentiation, and memory^7,8^.

In addition to regulating CAMK family members, CAMKK2 can phosphorylate AMP-activated protein kinase (AMPK). AMPK is a heterotrimeric protein complex that acts as an energy sensor and plays a crucial role in regulating cellular energy metabolism. Dysregulation of AMPK has been implicated in major chronic diseases, such as obesity, inflammation, diabetes, and cancer^9^. AMPK activity can be allosterically controlled via competitive binding of different adenine nucleotides (ATP, ADP, or AMP) to its regulatory gamma subunit^10,11^. Alternatively, AMPK activation can be triggered by CAMKK2 via a nucleotide-independent mechanism. Thus, CAMKK2 can decode increases in intracellular Ca^2+^ levels triggered by upstream extracellular events, such as insulin receptor binding, to activate AMPK and maintain energy levels^12–14^. Accordingly, CAMKK2-null mice are protected against weight gain induced by a high-fat diet, insulin resistance and glucose intolerance^13^.

The development of potent and selective small-molecule inhibitors to each of the CAMKKs would have a significant impact on our ability to investigate the roles these enzymes play in various biological processes. Due to the high degree of sequence and structure similarity between CAMKK1 and CAMKK2^15^, it is not surprising that most CAMKK inhibitors are equally potent against both enzymes^16^. Moreover, STO-609^17^, a commonly used inhibitor of CAMKKs, demonstrates IC_50_ values less than 250 nM for six other kinases when screened against a panel of only 92 protein kinases^18^. Due to this off-target activity, results obtained using STO-609 in a phenotypic assay cannot directly link the effect to CAMKK1-2. Thus, despite progress, the invention of a selective inhibitor for either CAMKK remains elusive.

Here we identified potent ligands to the kinase domain of CAMKK2 from a library of commercially available, small molecule kinase inhibitors. Enzyme assays confirmed most of our initial hits as inhibitors of both full-length CAMKK1 and CAMKK2. ITC data suggested that binding of the most potent compounds was enthalpy driven. We also obtained and analyzed co-crystal structures for the kinase domain of CAMKK2 bound to some of these compounds. We expect our data to assist the future development of inhibitors selective to each human CAMKK protein.

## Results and Discussion

### Identification of ligands that bind to CAMKK2

**Figure 1:**
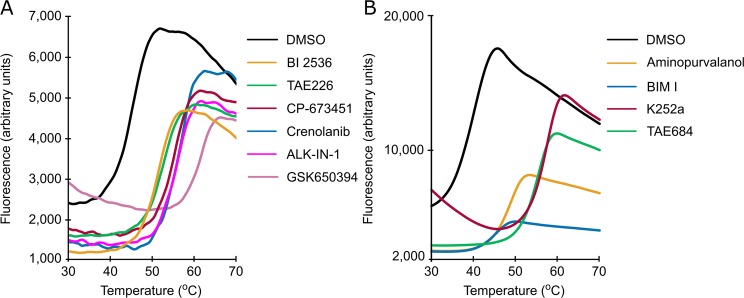
DSF screening identified ligands of CAMKK2-KD. Shown are CAMKK2-KD thermal melting curves in the presence of selected compounds or vehicle only (DMSO control) from (**A**) 384-well and (**B**) 96-well format assays. ΔTm values for these compounds can be found in Table 1.

We employed a thermal-shift assay (Differential Scanning Fluorimetry, DSF)^19^ to identify compounds that can bind to the kinase domain of CAMKK2 (CAMKK2-KD). Protein thermal denaturation curves (Fig. 1A,B) were used to calculate the denaturation midpoint temperature (Tm) for the kinase domain of CAMKK2 in the presence of ligands or vehicle only (DMSO). Changes in CAMKK2-KD Tm, ΔTm, were calculated by subtracting Tm values obtained in the presence of compounds from that observed in the presence of vehicle only. DSF screens were performed on purified CAMKK2-KD using a library of commercially available, chemically diverse, ATP-competitive, kinase inhibitors. Compounds were available from two different laboratories (SGC-UNICAMP and SGC-Oxford), and DSF screens utilized two different formats (384- and 96-well, respectively). These differences in assay format prevented a direct comparison of absolute ΔTm values found for compounds in each library. Nevertheless, compound ranking order obtained from either format was still informative. For example, two structurally related compounds from different libraries (staurosporine - 384-well format; and K252a - 96-well format) were found as top hits in both formats.

Table 1 shows ΔTm values for selected compounds (a comprehensive list can be found in Supplementary Table S1). Although not quantitative, DSF is a robust method to estimate binding affinities^19^. Indeed, we found a good agreement between our DSF ΔTm results and available *K*_D_ values from previously-published, unrelated binding^20^ and enzymatic assays^21^ using the full-length protein (Supplementary Table S2). For example, staurosporine, a pan-kinase inhibitor, displayed the largest ΔTm (17.1 °C) in our DSF assay and a *K*_D_ of 200 pM in the binding displacement assay. One notable discrepancy was Foretinib, a Type II kinase inhibitor initially developed to target members of the HGF and VEGF receptor tyrosine kinase families (see below)^22^. This compound displayed a high ΔTm in our DSF assay (6.3 °C) and a low *K*_D_ (4,400 nM) in the binding displacement assay^20^.

**Table 1:** Values obtained from DSF, enzymatic activity and ITC experiments.

Compound	CAMKK2-KD	CAMKK2-FL	CAMKK1-FL	CAMKK2-KD
	ΔTm (°C)^a^	IC_50_ (nM)^b^		ITC *K*_D_ (nM)^c^
Staurosporine	17.1^d^	5.0	19.0	—
GSK650394	16.2^d^	27.0	21.0	4.0
ALK-IN-1	11.2^d^	11.0	182.0	2.0
Crenolanib (CP-868596)	10.6^d^	21.0	82.0	—
CP-673451	10.0^d^	19.0	122.0	2.0
TAE226	7.5^d^	62.0	395.0	—
Foretinib	6.3^d^	1,304.0	200.0	—
BI 2536	6.1^d^	29.0	121.0	9.0
BI 6727	6.0^d^	44.0	87.0	—
Crizotinib	3.3^d^	3,907.0	>10,000	—
K252a	18.3^e^	50.0	51.0	—
TAE684	16.3^e^	21.0	56.0	—
Aminopurvalanol	9.7^e^	159.0	188.0	—
BIM I	7.2^e^	3,550.0	2,591.0	—

^a,b^Values shown are for single measurements; ^c^Values shown are for serial integrations of two independent experiments; ^d^384-well format; ^e^96-well format.

A non-exhaustive survey of the literature indicated that most of the compounds identified via DSF here have not previously been shown to bind CAMKK2-KD (Supplementary Table S2)^20,21^. All ΔTm values reported here were derived from a single experiment.

### Compounds identified using DSF inhibit the activity of both full-length CAMKK1 and CAMKK2

To confirm our top DSF results for CAMKK2-KD, we employed an enzymatic assay using the full-length protein (CAMKK2-FL). The enzymatic reaction was performed in the presence of Ca^2+^/Calmodulin. Potency values (IC_50_) can be found in Table 1 and inhibitor titration curves in Supplementary Fig. S1. All IC_50_ determinations described here were performed in singlets.

Data from the enzymatic assay largely corroborated findings from the DSF screen. Compounds inducing large shifts in melting temperature were potent inhibitors of CAMKK2-FL and displayed IC_50_ values ≤ 50 nM. These were: staurosporine, GSK650394, ALK-IN-1, Crenolanib, CP-673451, BI 2536, BI 6727, K252a and TAE684. Aminopurvalanol and TAE226, two compounds eliciting moderate to large ΔTms, displayed IC_50_ values of 159 and 62 nM, respectively. Three compounds identified in our DSF screen were not effective inhibitors of CAMKK2-FL (IC_50_ values >1,000 nM). These three compounds, BIM I, crizotinib and foretinib, are likely to be false positives. As mentioned above, foretinib is a Type II kinase inhibitor. In our experience, it is not unusual for Type II kinase inhibitors to be identified as binders in the DSF assay, but later fail to be active in an orthogonal assay, such as an enzymatic assay. However, we have not explored this issue systematically. Type I inhibitors BIM-I and crizotinib also failed to inhibit CAMKK2-FL. Data from a binding displacement assay had already shown crizotinib to be a weak binder of CAMKK2-FL^21^ (Supplementary Table S2). Currently, we do not have an explanation for the discrepancy observed between ΔTm and IC_50_ values for these compounds.

All compounds employed in this study were initially developed to target other human protein kinases, and some are known to be quite promiscuous^18,21^. Thus, we do not expect compounds identified here to be selective towards CAMKK2. To assist in the future development of selective CAMKK2 inhibitors, we also obtained IC_50_ values for CAMKK1-FL using the same compounds and assay conditions as for CAMKK2-FL above (Supplemental Fig. S2).

Not surprisingly, given the similarities between the two CAMKKs (see below), most assayed compounds were equipotent towards the two enzymes (Table 1). Notable exceptions were ALK-IN-1 and CP-673451. These two compounds were ~10-fold more active towards CAMKK2-FL than CAMKK1-FL.

### Molecular basis for ligand binding

To better understand the ligand-binding site of CAMKK2, we crystallized the protein kinase domain (amino acids 161–449) in the presence of some of the most potent CAMKK2-FL inhibitors - BI 2536, ALK-IN-1, GSK650394, CP-673451, TAE226, and Crenolanib. All co-structures were solved by molecular replacement using CAMKK2-STO-609^23^ as the search model (Table 2).

**Table 2:** Crystallographic data.

Ligand	ALK-IN-1	BI 2536	GSK650394
**Data collection**			
X-ray source	DLS I24	APS 24-ID-C	DLS I24
Wavelength (Å)	0.9686	0.9791	0.9686
Space group	P1	P4_3_2_1_2	P4_3_2_1_2
**Cell dimensions**			
*a, b, c* (Å) α, β, γ (°)	54.8, 54.9, 56.6 72.4, 78.4, 89.7	73.5, 73.5, 119.3 90.0, 90.0, 90.0	73.0, 73.0, 119.3 90.0, 90.0, 90.0
Resolution (Å)*	19.72–2.20 (2.27–2.20)	19.88–1.80 (1.84–1.80)	19.97–2.00 (2.05–2.00)
No. of unique reflections*	28,914 (2,482)	31,086 (1,798)	20,267 (1,601)
Rmerge (%)*	7.80 (32.6)	9.60 (115.0)	9.60 (172.4)
Mean I/σI * Mean CC(1/2)*	5.8 (1.3) 1.0 (0.5)	15.4 (2.2) 1.0 (0.8)	12.5 (1.4) 1.0 (0.5)
Completeness (%)*	92.8 (92.0)	99.9 (100)	89.9 (99.6)
Redundancy*	1.8 (1.8)	12.9 (12.8)	10.3 (10.4)
**Refinement**			
Resolution (Å)	19.73–2.20 (2.26–2.20)	19.88–1.80 (1.85–1.80)	19.97–2.00 (2.05–2.00)
Rcryst/Rfree (%)	18.2/22.1	19.1/22.8	19.3/23.7
**No. of atoms/Mean B-factor (Å)**			
Protein atoms	3,967/50.1	2,114/30.4	2,081/46.4
Solvent atoms	80/44.1	192/39.1	102/53.3
Ligand atoms	73/47.5	38/23.4	32/39.8
Rmsd bond lengths (Å)	0.010	0.011	0.009
Rmsd bong angles (degrees)	1.03	1.43	1.31
**Ramachandran statistics (%)**			
Favored	98.1	98.1	98.0
Allowed	1.9	1.9	2.0
Outlier	0	0	0
PDB ID	6BRC	6BQQ	6BKU
Crystallization conditions	26% PEG 3350, 0.2 M ammonium sulfate, 0.1 M SBG buffer, pH 6.0	22% PEG 3350, 0.21 M ammonium sulfate, 0.1 M CHC buffer, pH 7.5	20% PEG smear medium, 0.2 M sodium formate, 0.1 M sodium-potassium phosphate, 10% glycerol, pH 6.2
**Ligand**	**CP-673451**	**TAE-226**	**Crenolanib**
**Data collection**			
X-ray source	DLS I24	APS 24-ID-C	APS 24-ID-C
Wavelength (Å)	0.9686	0.9791	0.9791
Space group	P2_1_2_1_2_1_	P4_3_2_1_2	P2_1_2_1_2_1_
**Cell dimensions**			
*a, b, c* (Å) α, β, γ (°)	49.0, 77.7, 78.1 90.0, 90.0, 90.0	73.7, 73.7, 123.9 90.0, 90.0, 90.0	49.1, 77.8, 78.7 90.0, 90.0, 90.0
Resolution (Å)*	19.78–1.90 (1.94–1.90)	19.94–2.00 (2.05–2.00)	19.89–1.95 (2.00–1.95)
No. of unique reflections*	22,633 (1,224)	22,752 (1,687)	22,637 (1,565)
Rmerge (%)*	5.80 (93.1)	9.30 (100)	4.80 (89.9)
Mean I/σI * Mean CC(1/2)*	13.1 (1.8) 1.0 (0.6)	12.2 (2.1) 1.0 (0.7)	18.9 (1.9) 1.0 (0.8)
Completeness (%)*	93.6 (76.8)	96.5 (98.5)	99.8 (99.9)
Redundancy*	5.2 (4.8)	8.4 (8.6)	6.4 (6.7)
**Refinement**			
Resolution (Å)	19.78–1.90 (1.95–1.90)	19.94–2.00 (2.05–2.00)	19.89–1.95 (2.00–1.95)
Rcryst/Rfree (%)	18.6/22.7	18.7/22.5	18.9/22.7
**No. of atoms/Mean B-factor (Å)**			
Protein atoms	2,076/40.4	2,167/39.2	2,023/41.2
Solvent atoms	90/44.0	112/42.9	100/45.1
Ligand atoms	37/33.4	47/41.1	37/35.8
Rmsd bond lengths (Å)	0.009	0.014	0.015
Rmsd bong angles (degrees)	1.33	1.60	1.64
**Ramachandran statistics (%)**			
Favored	97.3	98.0	96.0
Allowed	2.7	2.0	4.0
Outlier	0	0	0
**PDB ID**	6BLE	6BQL	6BQP
**Crystallization conditions**	28% PEG 3350, 0.07 M ammonium acetate, 0.1 M SBG buffer pH 5.5	24% PEG 3350, 0.14 M ammonium acetate, 0.1 M CHC buffer pH 7.5	22% PEG 3350, 0.21 M ammonium acetate, 0.1 M CHC buffer pH 7.0

^*^Numbers in parenthesis indicate statistics for the highest resolution shell.

**Figure 2:**
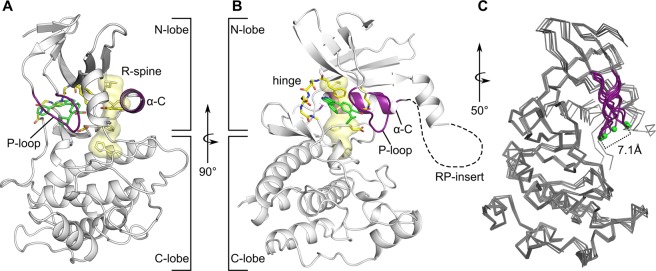
The overall structure of CAMKK2-KD bound to GSK650394 and overlay of all ligand-bound CAMKK2-KD structures from this work. (**A**,**B**) Cartoon representation of CAMKK2-KD showing the conserved kinase domain architecture consisting of N- and C-lobes connected by an intervening hinge region (main chain atoms shown as sticks). Major structural elements within the kinase domain are highlighted. The kinase domain α-C and P-loop are shown in purple. The R-spine is shown as a yellow surface, and side chains for residues within the R-spine are shown as sticks. Residues within the RP-insert are not part of the final model and this region is represented by a dashed black line. (**C**) Overlay of CAMKK2-KD structures (shown as ribbon) highlighting differences in the protein P-loop (in purple). Green spheres represent Cα atoms for residues Ser175 within the protein P-loop.

As expected, co-crystal structures of CAMKK2-KD bound to different ligands displayed the canonical kinase domain architecture: a smaller N-terminal lobe composed mostly of β-strands (residues 161 to 266) connected by a short hinge region (residues 267–273) to a larger C-terminal lobe made predominantly of α-helices (residues 274–449). The kinase domain ATP-binding site locates to a crevice between the two lobes, in which ATP-competitive inhibitors commonly bind (Fig. 2A,B - CAMKK2-KD bound to GSK650394). In all co-structures of CAMKK2-KD obtained here, the protein displayed an active conformation, in which the R-spine is fully formed (shown as a yellow surface in Fig. 2A,B). The R-spine is a set of 4 residues in kinases whose side chains line up to form a “spine” when the enzyme is in an active conformation^24^. In CAMKK2 these residues are Leu243, Phe331, His310, and Leu251 (Fig. 2). Our co-structures also presented other hallmarks of the kinase active conformation. Helix α-C was close to the protein ATP-binding site, an arrangement that allowed the side chains of conserved lysine (Lys194 in CAMKK2) and glutamate (Glu263 in CAMKK2) residues to interact. Side chains of residues within the conserved DFG motif were also found in positions characteristics of the kinase active conformation: the side chain of DFG residue Asp330 pointed towards the ATP-binding site, whereas that of DFG residue Phe331 was part of the R-spine. The final models for our co-structures lacked most of the region between β-3 and α-C (residues 206–229). This region is known as the RP-insert and is rich in proline (8 out of 24), glycine (5 out of 24) and arginine (5 out of 24) residues, and likely to be disordered (shown as a dashed line in Fig. 2B). The related human kinase, CAMMK1, also displays a similarly disordered region^15^.

An overlay of all six co-structures revealed that the overall structure of CAMKK2-KD bound to various ligands is mostly unchanged (root mean squared deviation - r.m.s.d. between Cα atoms ≤0.55 Å). The most notable exception to this observation concerned the position of the protein P-loop. The P-loop is an inherently flexible (GxGxF/YG conserved motif - residues 172–178 in CAMKK2) region of the kinase domain that folds over the phosphate atoms from the ATP molecule. For the different CAMKK2-KD co-structures obtained here, the distance between equivalent C-α atoms from P-loop residue Ser175 varied as much as 7.1 Å (Fig. 2C).

**Figure 3:**
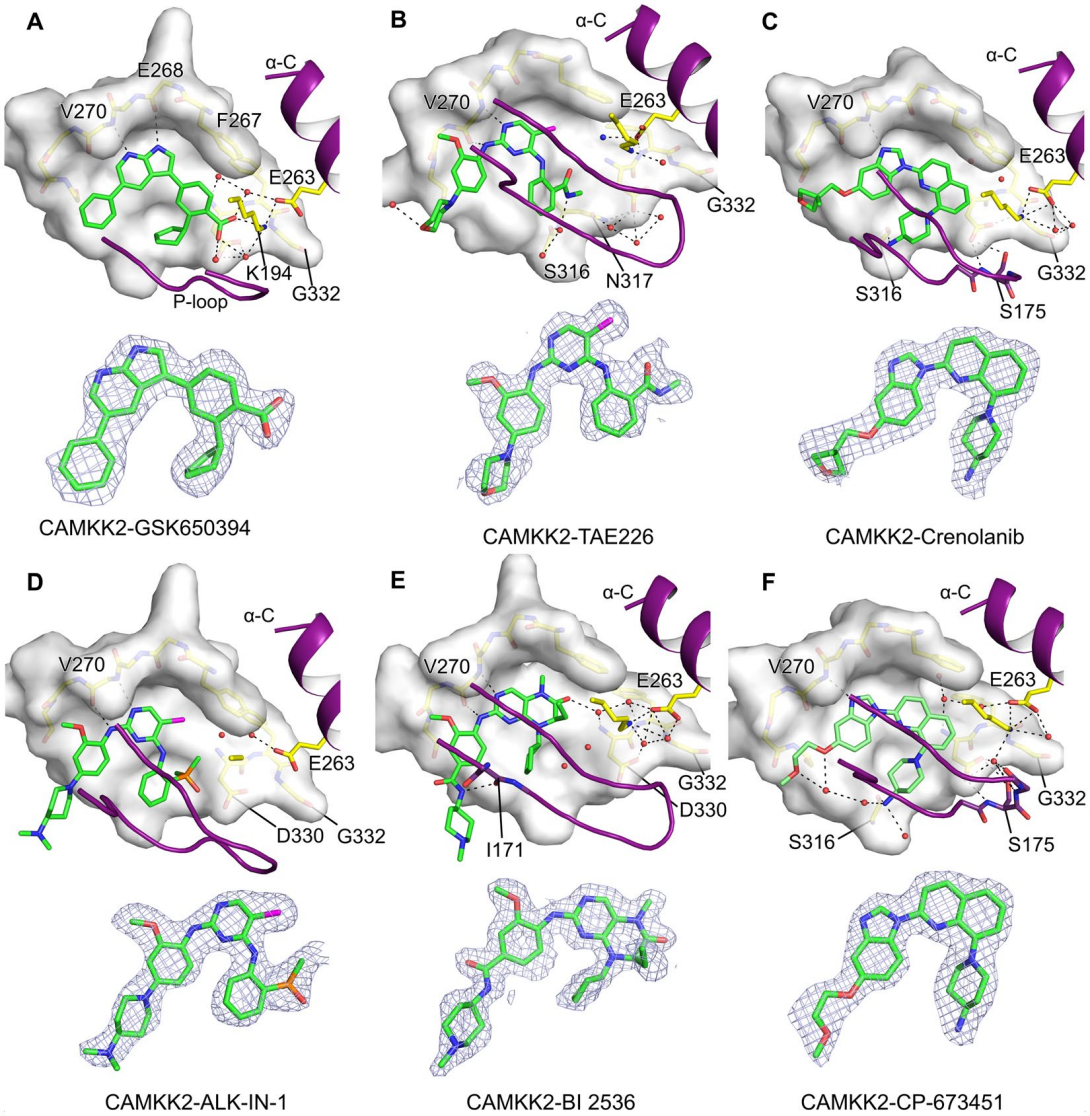
Binding mode of small-molecule ligands to CAMKK2-KD ATP-binding pocket. (**A**–**F**) The top part of each panel shows the ligand binding mode to CAMKK2-KD ATP-binding site (shown as molecular surface). Main chain atoms for residues (267–272) within CAMKK2-KD hinge region are represented as sticks. Side-chain atoms for CAMKK2-KD gatekeeper residue (Phe267) and structurally conserved lysine (Lys194) and glutamate (Glu263) residues are also shown as sticks. Black dashed lines represent possible hydrogen bonds between protein, crystallographic water molecules (shown as red spheres) and ligand atoms (shown as stick). The bottom part of each panel shows omit maps for each ligand calculated using SFCHECK^36^ within the CCP4 suite (contoured at 1.0 σ - gray meshes).

For all co-structures, electron density maps were of good quality and allowed the unambiguous building of the small molecule ligands within the protein ATP-binding site (Fig. 3A–F). All compounds were anchored to the protein kinase hinge region via a hydrogen bond to the main chain amide of residue Val270. For the majority of the compounds, an additional hydrogen bond was observed between the ligand and a carbonyl oxygen atom from the hinge region - Glu268 to GSK650394 or Val270 to TAE-226, ALK-IN-1 and BI 2536. The carbonyl group from residue Ser316 also mediated a hydrogen bond to *N*-containing moieties from Crenolanib and CP-673451. Similarly, the carbonyl group of residue Ile171 mediated a hydrogen bond to BI 2536 amide. Interactions between the various ligands and CAMKK2-KD are shown in Supplementary Fig. S3.

Interestingly, only GSK650394 occupied the region of CAMKK2 ATP-binding site between conserved residues Lys194 and Glu263, and this compound was the only one to directly engage the side chain of Lys194 via hydrogen bonds (Fig. 3A). Residue Phe267, which occupies the gate-keeper position within CAMKK2-KD ATP-binding site, mediated T-shaped π-π stacking interactions to aromatic rings in CP-673451, Crenolanib, and GSK650394. The same residue also mediated carbonyl-π (to BI 2536) or Cl-π (to TAE226 and ALK-IN-1) interactions to other ligands.

**Figure 4:**
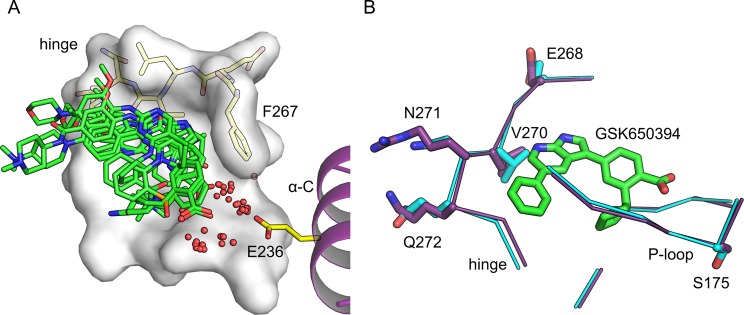
Crystallographic waters in CAMKK2-KD ATP-binding site and structural differences between CAMKK1-KD and CAMKK2-KD ATP-binding sites. (**A**) Top view of an overlay of CAMKK2-KD ATP-binding sites from all structures determined in this work plus those bound to STO-609 (PDB ID 2ZV2) and GSK650393 (PDB ID 6CMJ). Red spheres indicate solvent molecules found around the gatekeeper residue (Phe267) and the conserved glutamic acid in α-C (Glu236) from all analyzed CAMKK2-KD structures. The protein ATP-binding site is shown as molecular surface (as in Fig. 3). Ligands are depicted in stick model. Protein residues within the hinge region are shown as sticks. (**B**) Overlay of CAMKK1-KD (purple) and CAMKK2-KD (cyan) ATP-binding sites bound to GSK650394 (green). Side chains for divergent residues (labeled) between the two enzymes are shown as sticks.

We compared our CAMKK2-KD co-crystal structures to those obtained for the same protein bound to STO-609 (PDB ID 2ZV2)^23^ and a recently-developed azaindazole inhibitor similar to GSK650394 (PDB ID 6CMJ)^16^. An overlay of the ATP-binding site in these CAMKK2 co-structures revealed that future compounds could explore the addition of polar groups to take the place of the structural water molecules found trapped between the inhibitors and a conserved glutamate (Glu263) from the protein α-C helix (Fig. 4A). Using inhibitor atoms to satisfy polar interactions originally facilitated between protein groups and crystallographic water molecules has been used successfully to increase compound potency in some cases. For example, Kung and colleagues used this strategy to increase the potency of compounds targeting HSP90^25^.

### Structural differences between CAMKK1 and CAMKK2 ATP-binding sites

Despite the lack of a targeted medicinal chemistry effort, potent inhibitors for both CAMKK proteins do exist^16,17^, and a possible way forward to advance our understanding of the function of these two proteins might be the development of selective inhibitors of each enzyme. At the amino acid sequence level, CAMKK1-FL and CAMKK2-FL are ~60% identical. Nevertheless, the ATP-binding pockets of the two CAMKKs are highly similar. Analysis of the crystal structures of each one of the CAMKKs bound to compound GSK650394 revealed that out of the 26 residues within 5 Å of ligand atoms, only five (19%) differ between these two proteins (Fig. 4B). Four of these differences (Asp231 vs Glu268; Leu233 vs Val270; Arg234 vs Asn271 and Lys235 vs Gln272 - CAMKK1 vs CAMKK2) locate to the protein hinge region. These differences might prove challenging to exploit while designing selective compounds to each CAMKK, as the side chains of these four residues point away from the protein ATP-binding site. Another difference between the ATP-binding sites of the two CAMKKs locates to the P-loop of the protein (Ala138 vs Ser175 - CAMKK1 vs CAMKK2). Our structures suggest that this difference could be utilized to design compounds with increased potency towards CAMKK2 over CAMKK1 by the introduction of groups to facilitate polar interactions to the side chain hydroxyl group of CAMKK2 Ser175.

Recently, our group obtained the structure of CAMKK1-KD and proposed structural differences between the two CAMKKs that could be capitalized on for the design of selective molecules to each protein. One of these strategies included taking advantage of the extra space observed for hinge residue Val270 in CAMKK2 (Leu233 in CAMKK1)^15^. This suggestion was also put forward by Price and colleagues^16^.

### Favorable enthalpy dominates binding of most potent ligands to CAMKK2-KD

**Figure 5:**
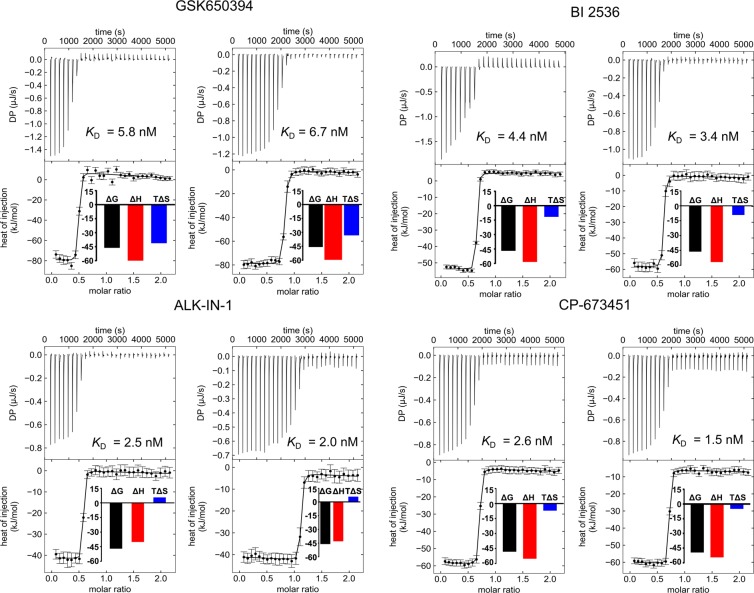
Dissociation constant measurements by ITC for selected compounds against CAMKK2-KD. The top part of each panel shows the injection heats, the bottom part shows the fitted binding isotherms (a single-site model was used), and the insets show the binding energies in kJ/mol.

Both DSF and enzyme inhibition assays described above were performed in singlets. We employed ITC to confirm these results and to characterize the thermodynamic parameters of ligand binding. All ITC experiments described below were performed twice. For all tested compounds, estimated binding affinities (*K*_D_) and thermodynamic parameters from both ITC runs were found to be in good agreement with each other (Table 3 and Fig. 5).

**Table 3:** Binding affinities and thermodynamic parameters for individual ITC experiments.

Compound	*K*_D_ (nM)		ΔG (kJ/mol)		ΔH (kJ/mol)		TΔS (kJ/mol.K)	
	*Run 1*	*Run 2*	*Run 1*	*Run 2*	*Run 1*	*Run 2*	*Run 1*	*Run 2*
GSK650394	5.8	6.7	−46.2	−45.8	−87.5	−79.1	−41.3	−33.2
ALK-IN-1	2.5	2.0	−46.0	−45.7	−43.0	−40.7	5.8	4.9
BI 2536	4.4	3.4	−47.5	−45.7	−58.0	−55.3	−10.5	−9.6
CP-673451	2.6	1.5	−48.2	−49.5	−55.2	−54.5	−7.1	−5.0

We found that top hits in the DSF - GSK650394, ALK-IN-1, CP-673451 and BI 2536; were all potent binders of CAMKK2 and had *K*_D_ values ranging from 1.5 to 6.7 nM (Table 3, and Fig. 5). In general, *K*_D_ values from ITC experiments were in good agreement with ΔTm and IC_50_ values obtained for the same compounds by DSF and enzymatic assays, respectively (Table 1).

Binding of all assayed compounds to CAMKK2-KD was enthalpy-driven, which is usually associated with the presence of hydrogen bonds and van der Waals interactions between ligand and protein^26,27^. Indeed, our crystallography data indicated that GSK650394, ALK-IN-1, CP-673451, and BI 2536 all engaged the protein hinge region via at least one hydrogen bond. These non-covalent interactions could account for the favorable enthalpy values observed in our ITC experiments (ranging from −87.5 to −43.0 kJ/mol - Table 3).

## Conclusion

In conclusion, we present here six new co-structures of commercial kinase inhibitors bound to CAMKK2-KD. Our co-structures demonstrated that all ligands had a similar binding conformation to CAMKK2-KD and revealed possible design strategies for the identification of more potent and selective CAMKK2 inhibitors. An inhibitor selective for CAMKK2 would be able to delineate the biological functions of CAMKK1 and CAMKK2 and further illuminate potentially unique roles CAMKK2 plays in disease, energy metabolism, and the nervous system.

## Methods

### Cloning, protein expression, and purification

Cloning, protein expression and purification for CAMKK2 kinase domain (CAKK2-KD) followed a protocol previously established at the SGC-UNICAMP and described elsewhere^15,28^. Briefly, for crystallization and ITC experiments using CAMKK2 kinase domain (CAMKK2-KD), we employed a construct of CAMKK2 isoform 7 (residues 161–449) (NCBI NP_001257415.1 – SGC construct CAMKK2B-cb002) in vector pNIC28-Bsa4. The construct was transformed into *Escherichia coli* BL21(DE3) cells that co-express λ-phosphatase and three rare tRNAs (plasmid pACYC-LIC+)^29^. Cells were cultured in TB medium containing 50 µg/mL kanamycin and 34 µg/mL chloramphenicol at 37 °C with shaking until the OD_600_ reached ~3 and then cooled to 18 °C for 1 hour. Isopropyl β-D-1-thiogalactopyranoside (IPTG) was added to a final concentration of 0.1 mM, and cultures were left overnight at 18 °C. Cells were harvested by centrifugation then suspended in 2x lysis buffer [1x lysis buffer is 50 mM HEPES buffer, pH 7.5, 0.5 M KOAc, 10% (v/v) glycerol, 50 mM each arginine/glutamate, 10 mM imidazole, 1.0 mM tris(2-carboxyethyl)phosphine (TCEP), Protease Inhibitor Cocktail Set VII (Calbiochem, 1/500 dilution)] and flash-frozen in liquid nitrogen. For purification, cell pellets were thawed and sonicated on ice. Polyethyleneimine (pH 7.5) was added to the lysate to a final concentration of 0.15% (w/v), and the sample was centrifuged at 53,000 × g for 45 min at 4 °C. The supernatant was loaded onto a Ni-Sepharose resin (GE Healthcare), and recombinant CAMKK2-KD was eluted stepwise in 1x lysis buffer with 300 mM imidazole. Removal of the hexahistidine tag was performed at 4 °C overnight using recombinant TEV (Tobacco Etch Virus) protease. CAMKK2-KD lacking the 6xHis tag was further purified using reverse affinity chromatography on Ni-Sepharose followed by gel filtration (Superdex 200 16/60, GE Healthcare). Protein in gel filtration buffer (10 mM HEPES, 500 mM KOAc, 1.0 mM TCEP, 5% [v/v] glycerol) was concentrated to 9 mg/mL (measured by UV absorbance in a NanoDrop spectrophotometer (Thermo Scientific) using the calculated molecular weight and estimated extinction coefficient) using 30 kDa molecular weight cut-off centrifugal concentrators (Millipore) at 4 °C. The concentrated protein was flash-frozen in a liquid nitrogen bath and stored at −80 °C until use.

### Crystallization, data collection, and structure determination

Crystallization of CAMKK2-KD bound to various small molecule inhibitors followed a protocol previously established at the SGC-UNICAMP and described elsewhere^28^. Briefly, kinase inhibitors (dissolved in 100% DMSO) were added to the protein in 3-fold molar excess and incubated on ice for approximately 30 minutes. The mixture was centrifuged at 21,000 × g for 10 minutes at 4 °C before setting up 150 nL volume sitting drops at three ratios (2:1, 1:1, or 1:2 protein-inhibitor complex to reservoir solution). Crystallization experiments were performed at 20 °C. Crystals were cryoprotected in mother liquor supplemented with 25–30% glycerol before flash-cooling in liquid nitrogen for data collection. Diffraction data were collected at 100 K at the Advanced Photon Source 24ID-C, and at the Diamond Light Source beamline I24. Data collection statistics and crystallization conditions can be found in Table 2.

Diffraction data were integrated with XDS^30^ and scaled using AIMLESS from the CCP4 software suite^31^. The structure was solved by molecular replacement using Phaser^32^ and the kinase domain of CAMKK2 as the search model (PDB ID 2ZV2)^23^. Refinement was performed using REFMAC5^33^, and Coot^34^ was used for model building. Structure validation was performed using MolProbity^35^. Omit maps were calculated using SFCHECK^36^ within the CCP4 suite.

### Differential scanning fluorimetry (DSF)

#### 384-well format

DSF experiments in 384-well format followed a protocol previously established at the SGC-UNICAMP and described elsewhere^15^. Briefly, CAMKK2-KD protein was screened against a library of 378 structurally diverse ATP-competitive kinase inhibitors available from Selleckchem (Houston, TX, United States; catalog No. L1200). Each well contained 20 μL of 1 μM kinase in 100 mM potassium phosphate pH 7.0, 150 mM NaCl, 10% glycerol and the Applied Biosystems Protein Thermal Shift dye at the recommended concentration of 1:1000.

The compounds, previously solubilized in DMSO, were used at 10 µM final concentration and 0.1% DMSO. Plates were sealed using optically clear films and transferred to a QuantStudio 6 qPCR instrument (Applied Biosystems). The fluorescence intensity was measured during a temperature gradient from 25 to 95 °C at a constant rate of 0.05 °C/s, and protein melting temperatures were calculated based on a Boltzmann function fitting to experimental data, as implemented in the Protein Thermal Shift Software (Applied Biosystems). Protein in 0.1% DMSO was used as a reference.

#### 96-well format

Each well contained 20 μL of 2 μM kinase in 10 mM HEPES pH 7.5 and 500 mM NaCl. SYPRO Orange dye (Invitrogen) was used in 5X as final concentration.

Compounds, previously solubilized in DMSO, were used at 12 µM final concentration and 2.5% DMSO. Plates were sealed using optically clear films and transferred to an Mx3005p RT-PCR machine (Agilent). The fluorescence intensity was measured during a temperature gradient from 25 to 95 °C at a constant rate of 0.05 °C/s, and protein melting temperatures were calculated based on a Boltzmann function fitting to experimental data (GraphPad Prism 7). Protein in 2.5% DMSO was used as a reference.

For both formats, experiments were performed in singlets. Compounds that caused a shift in melting temperature of the protein (ΔTm) of 2 °C or higher compared to the reference were considered positive hits. The commercial source for all compounds can be found in Supplementary Table S1.

### Enzymatic assays and determination of IC_50_ values

Values for half-maximal inhibitory concentration (IC_50_) of various compounds were determined using a radiometric assay performed by Eurofins Discovery (MO, USA) according to the company’s protocols and briefly described below. Reaction conditions for assays using CAMKK1-FL were: 8 mM MOPS pH 7.0, 0.2 mM EDTA, 0.33 mg/mL MBP (myelin basic protein), 0.5 mM calcium chloride, 0.016 mg/ml calmodulin, 10 mM magnesium acetate and [gamma-^33^P-ATP] (200 µM). Reaction conditions for assays using CAMKK2-FL were: 20 mM Tris-HCl pH 8.5, 0.2 mM EDTA, 0.5% BSA, 0.5 mM calcium chloride, 0.016 mg/mL calmodulin, 150 µM peptide substrate (LSNLYHQGKFLQTFCGSPLYRRR), 10 mM magnesium acetate and [gamma^−33^P]-ATP (90 µM). The reactions were initiated by the addition of a Mg^2+^-ATP mixture. After incubation for 40 minutes at room temperature, the reaction was stopped by the addition of phosphoric acid to a final concentration of 0.5%. 10 µl of the reaction was then spotted onto a P30 filtermat and washed four times for 4 minutes in 0.425% phosphoric acid and once in methanol prior to drying and scintillation counting. Following subtraction of the average inhibitor control well counts, results were expressed as a percentage of the mean kinase activity in the positive control sample. The assay was performed in singlicate at 9 concentrations ranging from 10 µM to 0.001 µM allowing for an IC_50_ determination.

### Isothermal titration calorimetry

Measurements were made using a MicroCal AutoITC200 (Malvern, United Kingdom). Purified CAMKK2-KD was dialyzed into gel filtration buffer (20 mM Hepes, pH 7.5, 300 mM NaCl, 5% glycerol, and 1 mM TCEP - overnight at 4 °C). Small molecule inhibitors were diluted in dialysis buffer. All ITC experiments were performed using a “reverse titration” setup in which the protein (in the syringe) was titrated into the cell containing the inhibitor. Two independent ITC measurements were performed for each compound, data for individual runs were integrated separately to obtain single isotherms, which were plotted and analyzed using Sedphat^37^. For BI 2536, CAMKK2-KD was used at 106.8 µM (run 1) and at 130 µM (run 2); the inhibitor was used at 10.7 µM (run 1) and at 13 µM (run 2). For ALK-IN-1, CAMKK2-KD was used at 105.6 µM (run 1) or at 106.6 µM M (run 2); the inhibitor was used at 10.6 µM (run 1) and at 10.7 µM (run 2). For GSK650394, CAMKK2-KD was used at 130 µM (run 1) or at 105.8 µM (run 2); the inhibitor was used at 13 µM (run 1) and at 10.6 µM (run 2). For CP-673451, CAMKK2-KD was used at 105.8 µM and the inhibitor was used at 10.6 µM in both runs. All measurements were made at 20 °C with mixing (1,000 rpm stirring), and used 1.5 µL injections and 180 s between each injection. NITPIC, SEDPHAT and GUSSI^37^ were used to analyze and generate figures for ITC data.

## Supplementary information

Supplementary Information

## Data Availability

The coordinates and structure factors for all CAMKK2-KD co-crystal structures reported here have been deposited in the Protein Data Bank with accession codes 6BRC, 6BQQ, 6BKU, 6BLE, 6BQL, and 6BQP.
