# Supplementary material for: Binding and structural analyses of potent inhibitors of the human Ca2+/calmodulin dependent protein kinase kinase 2 (CAMKK2) identified from a collection of commercially-available kinase inhibitors
Source: Sci Rep. 2019 Nov 11;9:16452. doi: 10.1038/s41598-019-52795-1 (PMC6848146; doi:10.1038/s41598-019-52795-1)
Supplement: Supplementary file 1 — Supplementary Information [file 41598_2019_52795_MOESM1_ESM.docx]

**Binding and structural analyses of potent inhibitors of the human Ca^2+^/calmodulin dependent protein kinase kinase 2 (CAMKK2) identified from a collection of commercially-available kinase inhibitors**

Gerson S. Profeta ^1,2^, Caio V. dos Reis ^1,2^, André da S. Santiago ^1,2^, Paulo H. C. Godoi ^1,2^, Angela M. Fala ^1,2^, Carrow I. Wells ^4^, Roger Sartori ^2^, Anita P. T. Salmazo ^2^, Priscila Z. Ramos ^1,2^, Katlin B. Massirer ^1,2^, Jonathan M. Elkins ^2,3^, David H. Drewry ^4^, Opher Gileadi ^3^ & Rafael M. Couñago ^1,2 *^

1. Centro de Química Medicinal (CQMED), Centro de Biologia Molecular e Engenharia Genética (CBMEG), Universidade Estadual de Campinas (UNICAMP), Campinas, SP, 13083-875, Brazil

2. Structural Genomics Consortium, Departamento de Genética e Evolução, Instituto de Biologia, UNICAMP, Campinas, SP, 13083-886, Brazil

3. Structural Genomics Consortium, University of Oxford, Old Road Campus Research Building, Roosevelt Drive, Oxford, OX3 7DQ, UK

4. Structural Genomics Consortium, UNC Eshelman School of Pharmacy, University of North Carolina at Chapel Hill, Chapel Hill, NC 27599, USA

* Electronic address: rafael.counago@unicamp.br

**Supplementary Information**

**Supplementary Figure Legends**

**Supplementary Figure S1** - **IC_50_ curves for indicated compounds against CAMKK2-FL**. Solid circles indicate enzyme activity relative to control (DMSO vehicle only) at indicated compound concentrations. Data was analyzed in GraphPad PRISM version 7. Calculated IC_50_ are shown in Table 1.

**Supplementary Figure S2** - **IC_50_ curves for indicated compounds against CAMKK1-FL**. Solid circles indicate enzyme activity relative to control (DMSO vehicle only) at indicated compound concentrations. Data was analyzed in GraphPad PRISM version 7. Calculated IC_50_ are shown in Table 1. All data shown are for single measurements.

**Supplementary Figure S3 - 2-D diagram showing potential polar interactions between different ligands and CAMKK2-KD**. Diagrams were generated using PoseView within the Proteins Plus web portal. Polar interactions between protein and ligand are drawn as dashed lines. Hydrophobic contacts are represented by green splines. Contacting amino acids are indicated.

| **Supplementary Table S1 - DSF screening results for compounds that increased the Tm of CAMKK2-KD > 4.0 °C.** | | | | |
| --- | --- | --- | --- | --- |
| **Compound** | **ΔTm (°C)**^c^ | | **Source** |  |
| Staurosporine ^a^ | 17.1 | SelleckChem | | |
| GSK650394 ^a^ | 16.2 | SelleckChem | | |
| ALK-IN-1 ^a^ | 11.2 | SelleckChem | | |
| Crenolanib (CP-868596) ^a^ | 10.6 | SelleckChem | | |
| CP-673451 ^a^ | 10.0 | SelleckChem | | |
| Ponatinib ^a^ | 9.8 | SelleckChem | | |
| AZD3463 ^a^ | 9.3 | SelleckChem | | |
| LDK378 ^a^ | 8.0 | SelleckChem | | |
| Pacritinib (SB1518) ^a^ | 7.8 | SelleckChem | | |
| TAE226 ^a^ | 7.5 | SelleckChem | | |
| Hesperadin ^a^ | 7.3 | SelleckChem | | |
| PIK-75 ^a^ | 7.2 | SelleckChem | | |
| Milciclib (PHA-848125) ^a^ | 7.0 | SelleckChem | | |
| GNE-9605 ^a^ | 6.4 | SelleckChem | | |
| Foretinib ^a^ | 6.3 | SelleckChem | | |
| LY2835219 ^a^ | 6.2 | SelleckChem | | |
| BI 2536 ^a^ | 6.1 | SelleckChem | | |
| Volasertib (BI 6727) ^a^ | 6.0 | SelleckChem | | |
| BX-795 ^a^ | 5.1 | SelleckChem | | |
| Danusertib ^a^ | 5.1 | SelleckChem | | |
| PHA-665752 ^a^ | 4.9 | SelleckChem | | |
| Pelitinib ^a^ | 4.8 | SelleckChem | | |
| WZ8040 ^a^ | 4.8 | SelleckChem | | |
| A-674563 ^a^ | 4.7 | SelleckChem | | |
| CCT137690 ^a^ | 4.7 | SelleckChem | | |
| BMS-777607 ^a^ | 4.7 | SelleckChem | | |
| TAK-901 ^a^ | 4.7 | SelleckChem | | |
| PF-573228 ^a^ | 4.5 | SelleckChem | | |
| Sunitinib Malate ^a^ | 4.5 | SelleckChem | | |
| Ro3280 ^a^ | 4.5 | SelleckChem | | |
| Flavopiridol HCl ^a^ | 4.5 | SelleckChem | | |
| CO-1686 ^a^ | 4.4 | SelleckChem | | |
| PF-3758309 ^a^ | 4.4 | SelleckChem | | |
| CYC116 ^a^ | 4.4 | SelleckChem | | |
| GSK1059615 ^a^ | 4.4 | SelleckChem | | |
| AZ 960 ^a^ | 4.3 | SelleckChem | | |
| LY2784544 ^a^ | 4.3 | SelleckChem | | |
| CYT387 ^a^ | 4.3 | SelleckChem | | |
| HS-173 ^a^ | 4.2 | SelleckChem | | |
| PF-562271 ^a^ | 4.2 | SelleckChem | | |
| AZD9291 ^a^ | 4.1 | SelleckChem | | |
| K252a ^b^ | 18.3 | LC Laboratories | | |
| TAE684 ^b^ | 16.3 | SelleckChem | | |
| Aminopurvalanol ^b^ | 9.7 | Calbiochem (EMD) | | |
| Syk Inhibitor ^b^ | 9.4 | Calbiochem (EMD) | | |
| AZD 7762 ^b^ | 8.4 | Axon Ligands | | |
| Wee1/Chk1 inhibitor ^b^ | 8.3 | Calbiochem (EMD) | | |
| BIM IX ^b^ | 8.1 | AXXORA | | |
| Flt3 Inhibitor III ^b^ | 7.5 | Calbiochem (EMD) | | |
| BioFocus 229-4051-4145 ^b^ | 7.2 | BioFocus | | |
| BIM I ^b^ | 7.2 | AXXORA | | |
| Dovitinib ^b^ | 6.9 | LC Laboratories | | |
| IKK 16 ^b^ | 6.8 | Tocris | | |
| NG 52 (Compound 52) ^b^ | 6.8 | Calbiochem (EMD) | | |
| JNK Inhibitor II ^b^ | 6.8 | Calbiochem (EMD) | | |
| GW806742X ^b^ | 5.8 | GSK | | |
| Indirubin-3monoxime ^b^ | 5.4 | Calbiochem (EMD) | | |
| Chk2 inhibitor II ^b^ | 5.4 | Calbiochem (EMD) | | |
| BioFocus 184-7897-0069 ^b^ | 5.1 | BioFocus | | |
| SU9516 ^b^ | 5.0 | Calbiochem (EMD) | | |
| GSK978744A ^b^ | 4.9 | GSK | | |
| T5694244 ^b^ | 4.8 | Enamine | | |
| JAK Inhibitor I ^b^ | 4.7 | Calbiochem (EMD) | | |
| Reversine ^b^ | 4.6 | Calbiochem (EMD) | | |
| BioFocus 184-7897-4140 ^b^ | 4.6 | BioFocus | | |
| ChemDiv C285-0110 ^b^ | 4.4 | ChemDiv | | |
| CID 755673 ^b^ | 4.4 | Tocris | | |
| Cdk2 Inhibitor III ^b^ | 4.4 | Calbiochem (EMD) | | |
| Aurora/Cdk Inhibitor ^b^ | 4.3 | Calbiochem (EMD) | | |
| BioFocus 184-0049-7467 ^b^ | 4.3 | BioFocus | | |
| Bosutinib isomer ^b^ | 4.1 | LC Laboratories | | |
| BioFocus 190-0053-4147 ^b^ | 4.1 | BioFocus | | |
| ChemDiv C285-0104 ^b^ | 4.1 | ChemDiv | | |
| BioFocus 184-4051-0074 ^b^ | 4.0 | BioFocus | | |

a - 384-well format; b - 96-well format; c - values reported from single measurements

| **Supplementary Table S2 - Literature Data for Binding and Enzymatic Assays Using Compounds Shown in Table1 Against CAMKK1 and CAMKK2** | | | | |
| --- | --- | --- | --- | --- |
| **Compound** | **% Activity Remaining**  **at 0.5 µM ^a^** | | **Binding results**  **(Kd's in nM) ^b^** | |
|  | **CAMKK2** | **CAMKK1** | **CAMKK2** | **CAMKK1** |
| Staurosporine | 0.24 | -3.13 | 0.16 | 0.039 |
| GSK650394 | N/T | N/T | N/T | N/T |
| ALK-IN-1 | N/T | N/T | N/T | N/T |
| Crenolanib (CP-868596) | N/T | N/T | N/T | N/T |
| CP-673451 | N/T | N/T | N/T | N/T |
| TAE226 | N/T | N/T | N/T | N/T |
| Foretinib | N/T | N/T | N/T | N/T |
| BI 2536 | N/T | N/T | 23 | 22 |
| BI 6727 | N/T | N/T | N/T | N/T |
| Crizotinib | N/T | N/T | 1,500 | >10,000 |
| K252a | N/T | N/T | N/T | N/T |
| TAE684 | N/T | N/T | 11 | 50 |
| Aminopurvalanol | 33.3 | 86.8 | N/T | N/T |
| BIM I | N/T | N/T | N/T | N/T |

a - Anastassiadis et al. (2011); b - Davis et al. (2011); N/T - not tested

**Supplementary Figure S1**

**
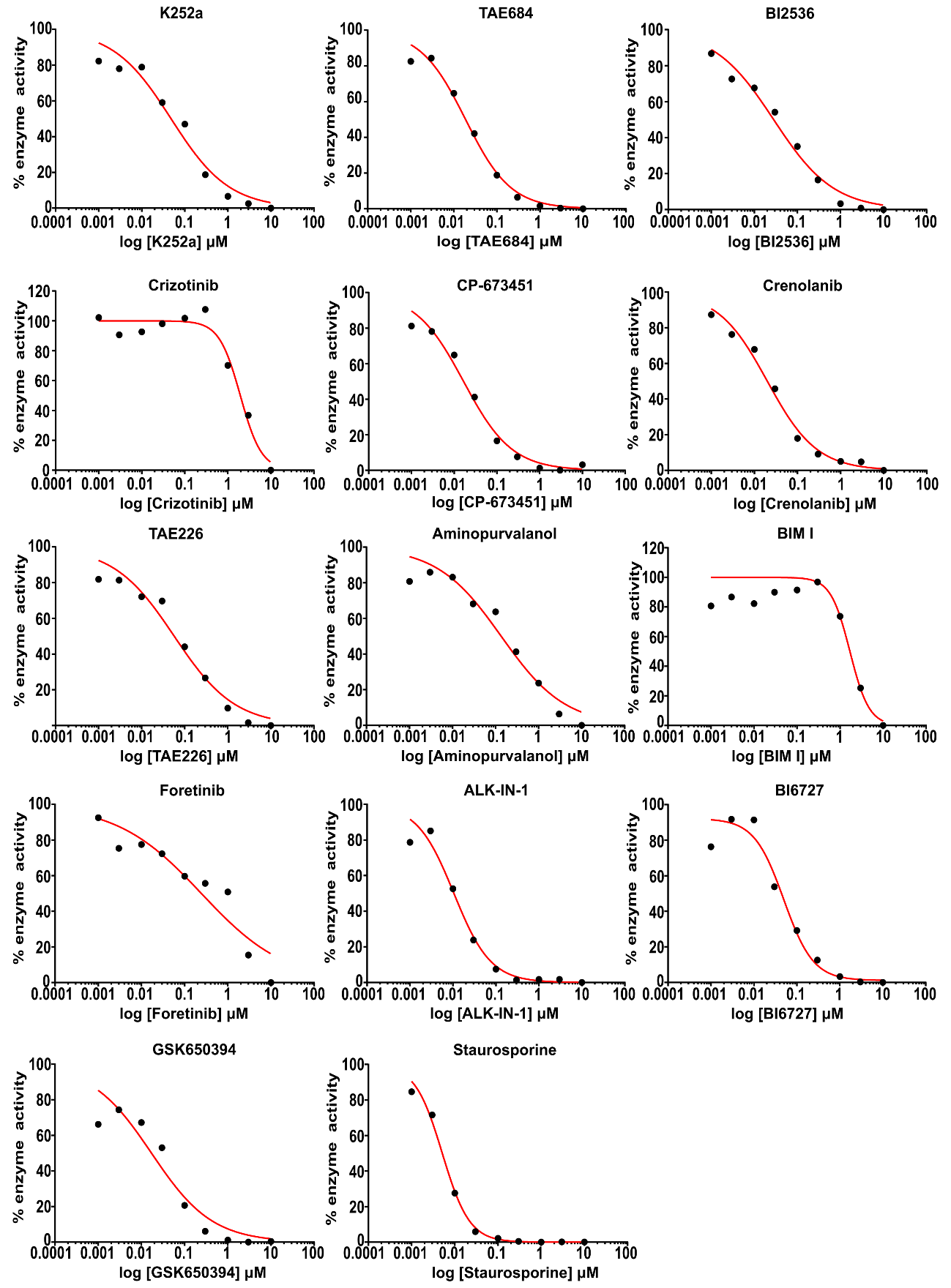
**

**Supplementary Figure S2**

**
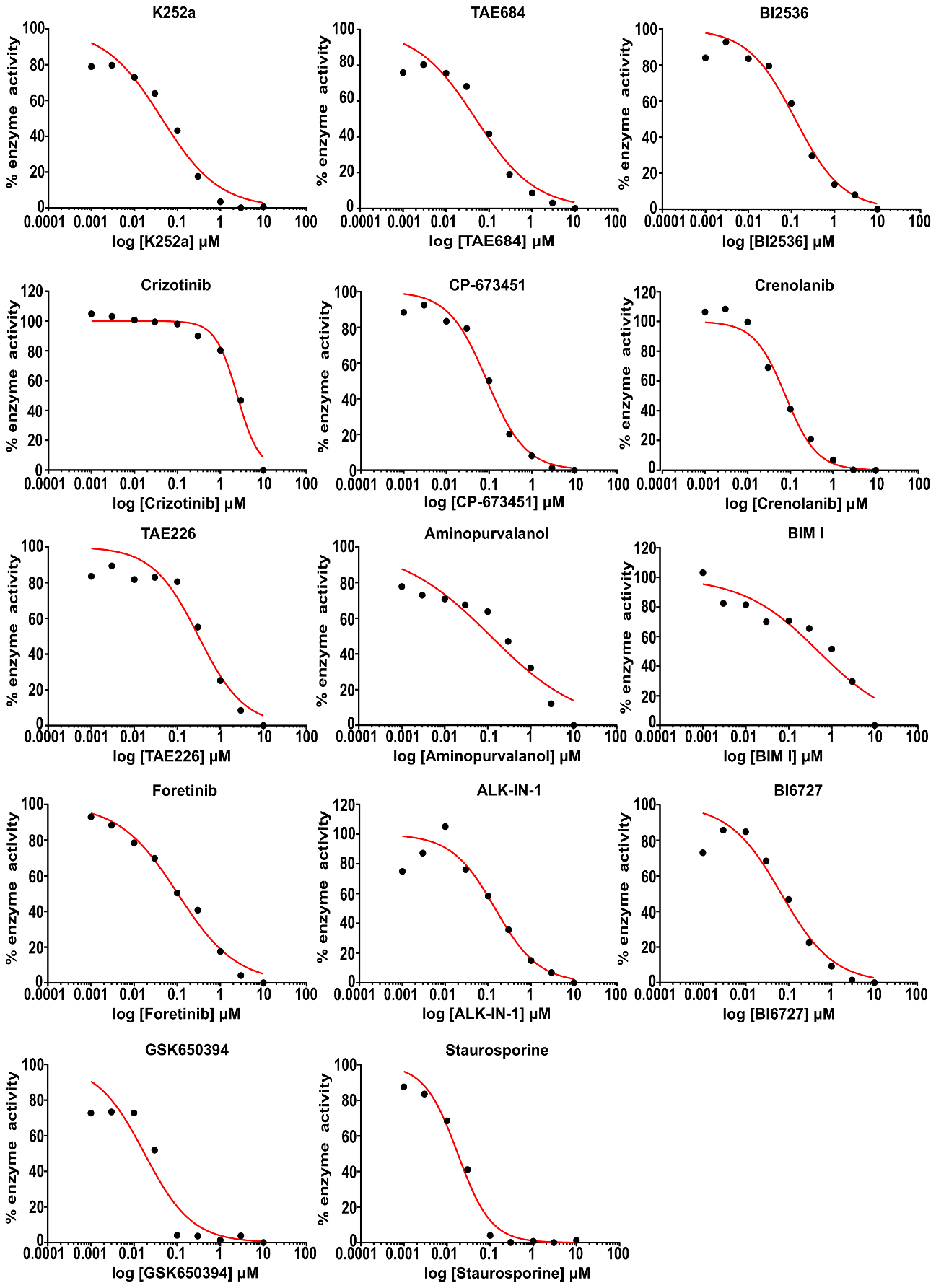
**

**Supplementary Figure S3**

**
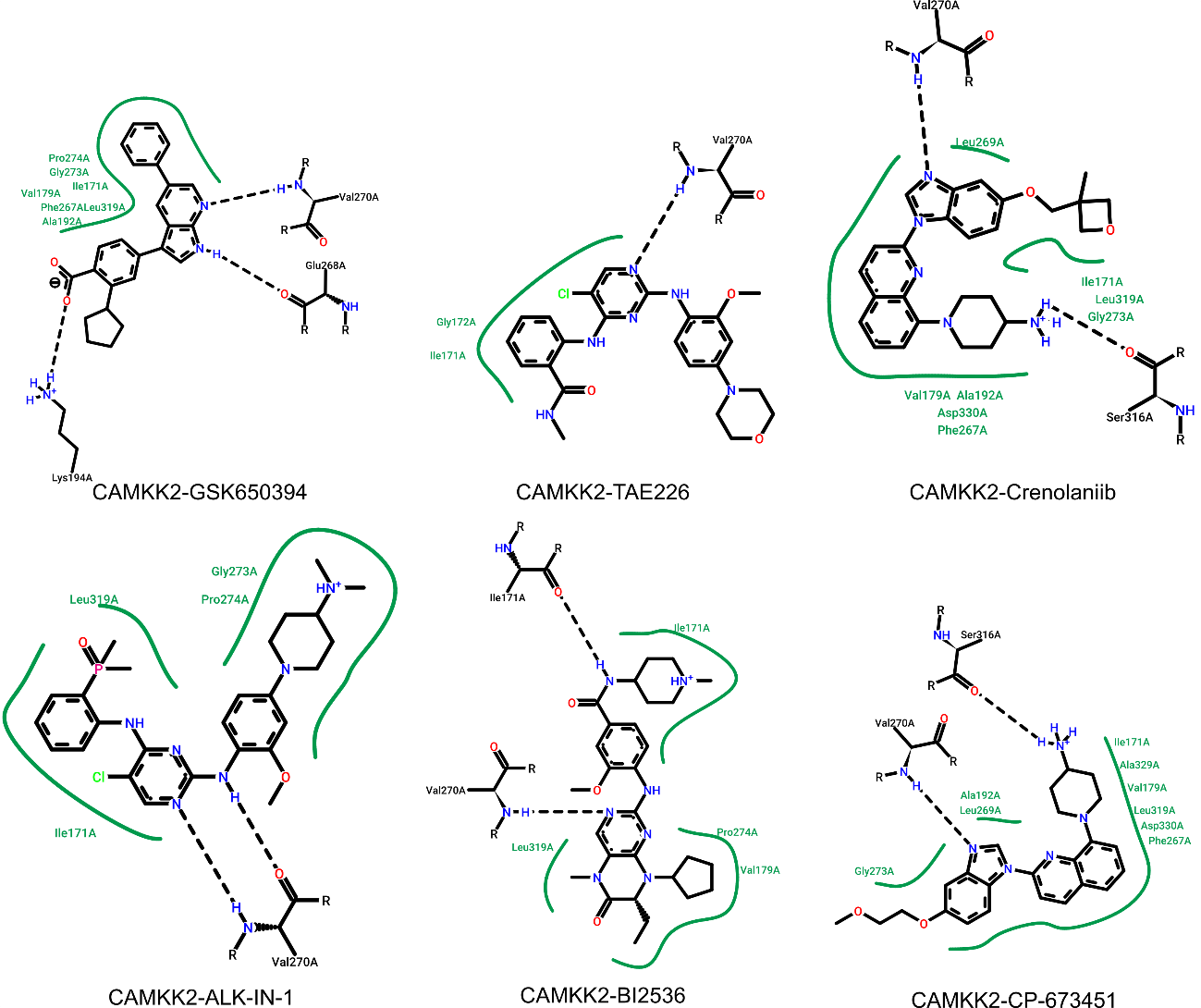
**
